# Clinical study on basal blood perfusion in the major arteries of the limbs

**DOI:** 10.3389/fmed.2025.1597404

**Published:** 2025-07-30

**Authors:** Rongji Zhang, Hao Wang, Ji Shi, Minghan Gao, Jianhui Li, Jianzheng Zhang

**Affiliations:** ^1^Graduate Division, Hebei North University, Zhangjiakou, China; ^2^Department of Orthopedics, Chinese PLA General Hospital, Beijing, China; ^3^Department of Hepatobiliary and Pancreatic Surgery, Shulan (Hangzhou) Hospital, Hangzhou, China

**Keywords:** color Doppler ultrasound, arteries, limb replantation, hemodynamic parameters, perfusion

## Abstract

**Background:**

Investigating basal blood perfusion in the major arteries of the limbs to guide flow rate selection during *ex vivo* perfusion preservation for limb replantation (transplantation).

**Methods:**

Volunteers undergoing physical examinations at PLAGH Fourth Medical Center (February–December 2024) were recruited. Three hundred and four eligible participants (146 males, 158 females; aged 18–65, mean 41.68 ± 11.28) were enrolled after screening. A portable Doppler ultrasound system was utilized to assess the brachial, ulnar, radial, popliteal, dorsalis pedis, and posterior tibial arteries in the limbs. Vascular diameter, blood flow velocity, and blood perfusion volume were measured for each artery. Mean hemodynamic parameters were calculated. Analyze the linear relationship between BMI, body surface area, and arterial blood perfusion volume using a multiple linear regression model. Conduct interaction tests to investigate whether there are sex-specific or “three-high” status-specific effects in the linear prediction model of blood perfusion volume by BMI and body surface area.

**Results:**

Ultrasound findings revealed the following mean blood perfusion volumes in the upper limb arteries: brachial artery, 74.9 ± 22.5 mL/min; ulnar artery, 35.7 ± 12.6 mL/min; radial artery, 36.8 ± 13.5 mL/min. In the lower limb arteries, the mean blood perfusion volume was: popliteal artery, 114.1 ± 34.2 mL/min; dorsalis pedis artery, 53.3 ± 18.1 mL/min; posterior tibial artery, 59.2 ± 21.0 mL/min. No significant difference was observed in mean blood perfusion volume between the ulnar and radial arteries (*p* > 0.05), whereas the posterior tibial artery exhibited significantly higher blood perfusion volume compared to the dorsalis pedis artery (*p* < 0.001). Multiple linear regression analysis revealed that BMI was negatively associated with arterial blood perfusion volume in the extremities, while body surface area showed a positive association. Furthermore, BMI and body surface area jointly formed a linear predictive relationship with limb blood flow. Based on significant effects within the linear model and pathophysiological mechanisms, interaction terms for body surface area (BSA) × BMI and age × diabetes status were included. The results demonstrated a statistically significant interaction effect (*p* < 0.05) between BMI and body surface area on limb blood flow. However, the interaction effect of diabetes status on limb blood flow was not significant (*p* > 0.05).

**Conclusion:**

Quantitative ultrasound-derived limb perfusion parameters and their BMI/BSA correlations enable hemodynamic customization for machine perfusion systems in limb replantation. This standard approach balances metabolic support and ischemia-reperfusion risk mitigation during extracorporeal preservation, advancing personalized transplant protocols.

## Introduction

Traumatic limb amputation, a severely disabling injury, has become one of the leading causes of limb loss (1). Patients not only face functional impairment of the affected limb but may also develop psychological disorders due to abrupt shifts in social roles and identity (2). Limb replantation and transplantation via microsurgical techniques, which achieve anatomical reconstruction and functional recovery of amputated limbs, not only represent the definitive approach for restoring patients’ motor function and aesthetic integrity but also serve as a cornerstone of clinical care to mitigate long-term disability rates and reduce the societal healthcare burden. However, the success of replantation surgery critically hinges on the effective preservation of the amputated limb and restoration of microcirculation. Clinically, muscle ischemia lasting 3 h induces necrosis, and functional recovery of the replanted limb declines to 3% of normal controls after 6 h of ischemia (3). Therefore, prolonging the effective preservation time of severed limbs has become a critical challenge in enhancing the success rate of replantation surgery. Traditional severed limb preservation has predominantly relied on static cold storage. While this method temporarily slows tissue metabolism, it fails to sustain cellular energy supply or facilitate metabolic waste clearance, inevitably inducing ischemia-reperfusion injury (IRI) post-replantation. Furthermore, static cold storage fails to maintain muscle structure and strength, providing no significant contribution to the functional restoration of replanted limbs post-surgery (4). Recent breakthroughs in limb perfusion techniques have opened new avenues to address this longstanding challenge. In contrast to static cold storage, perfusion technology has been clinically proven to dynamically circulate blood, replicating physiological hemodynamic conditions to deliver restorative benefits for marginal organs. This approach ensures functional viability to meet transplantation criteria (5). Furthermore, perfusion techniques can extend *ex vivo* organ survival time to 24 h (6, 7), buying critical time for patient transfer to specialized centers for replantation or transplantation. However, the clinical translation of this technology faces a critical barrier: the lack of evidence-based protocols for personalized perfusion parameterization. Current perfusion predominantly relies on animal-derived models or empirical settings (8–10), with insufficient investigation into physiological heterogeneity in human limb perfusion—particularly regarding individual variations in BSA, and BMI. This “one-size-fits-all” perfusion strategy risks under perfusion in low-flow zones, leading to ischemic injury and metabolic acidosis, while excessive flow in high-flow regions may damage vascular endothelium, exacerbating edema and inflammation—ultimately compromising therapeutic efficacy (11). Current literature has yet to establish personalized hemodynamic profiling of human blood flow, resulting in significant limitations when extrapolating experimental findings to clinical applications.

Addressing these challenges, this study pioneers the systematic quantification of resting-state basal blood flow in adult limbs using Doppler ultrasound. By constructing a multidimensional regression model integrating BMI, BSA, and limb blood flow, we elucidate the physiological principles governing hemodynamic distribution in human extremities. Building on this foundation, we propose a personalized blood perfusion volume algorithm that provides an evidence-based medical framework for limb preservation techniques. This advancement enables a paradigm shift from empirical perfusion protocols to standard-guided strategies, ultimately enhancing clinical outcomes in limb replantation and transplantation through optimized hemodynamic management.

## Materials and methods

### Study subjects

Inclusion criteria: (1) Individuals undergoing routine health examinations at our institution between January 2024 and December 2024; (2) Aged 18–65 years; (3) Voluntary participation with informed consent from both participants and their legal guardians. Exclusion criteria: (1) Psychiatric disorders: Patients diagnosed by a psychiatrist according to ICD-10 criteria with conditions causing communication or cooperation impairment (e.g., schizophrenia, bipolar disorder, major depressive episode). (2) Limb vascular abnormalities: (a) Anatomical variations: Congenital variants identified on ultrasound (e.g., persistent median artery, high radial artery bifurcation, aberrant arterial origins, popliteal artery entrapment syndrome). (b) Pathological abnormalities: Conditions confirmed by prior imaging/surgery (e.g., arteriovenous fistula, vascular aneurysm, traumatic vessel transection, history of vascular reconstruction surgery). (3) Pregnancy or lactation: Confirmed by serum β-hCG testing. (4) Incomplete clinical data: Missing essential records (e.g., complete ultrasound parameters, medication history). (5) Recent use of relevant medications: Vasoactive drugs (including nitrates, calcium channel blockers) Anticoagulant/antiplatelet agents (e.g., warfarin, aspirin) within the pre-test period.

### General characteristics

From January to December 2024, 623 individuals undergoing health examinations at the Fourth Medical Center of the PLA General Hospital were initially enrolled. Following the application of inclusion/exclusion criteria, 304 participants (146 male, 158 female; aged 18–65 years, mean ± SD: 41.68 ± 11.28 years) were included in this study. Height and weight were meticulously measured to calculate body mass index (BMI) and BSA. BMI calculation formula: BMI (kg/m^2^) = $\frac{W}{{\left(\frac{H}{100}\right)}^{2}}$ ; BSA calculation formula (12): BSA (m^2^) = $\frac{71.3989\times H^{0.7437}\times W^{0.4040}}{10,000}$ ; where *H* denotes height in centimeters (cm) and *W* denotes weight in kilograms (kg). This observational study was approved by the Ethics Committee of the Fourth Medical Center of the General Hospital of the Chinese People’s Liberation Army (Approval Number: 2021KY058-HS001), with written informed consent obtained from all participants.

### Methods

#### Equipment

The Mindray M9 portable color Doppler ultrasound system, equipped with an L12-4s linear array probe (frequency range: 3–13 MHz), was operated by two board-certified sonographers with extensive clinical experience and specialized training in the study protocol.

#### Inspection methods

**Figure 1:**
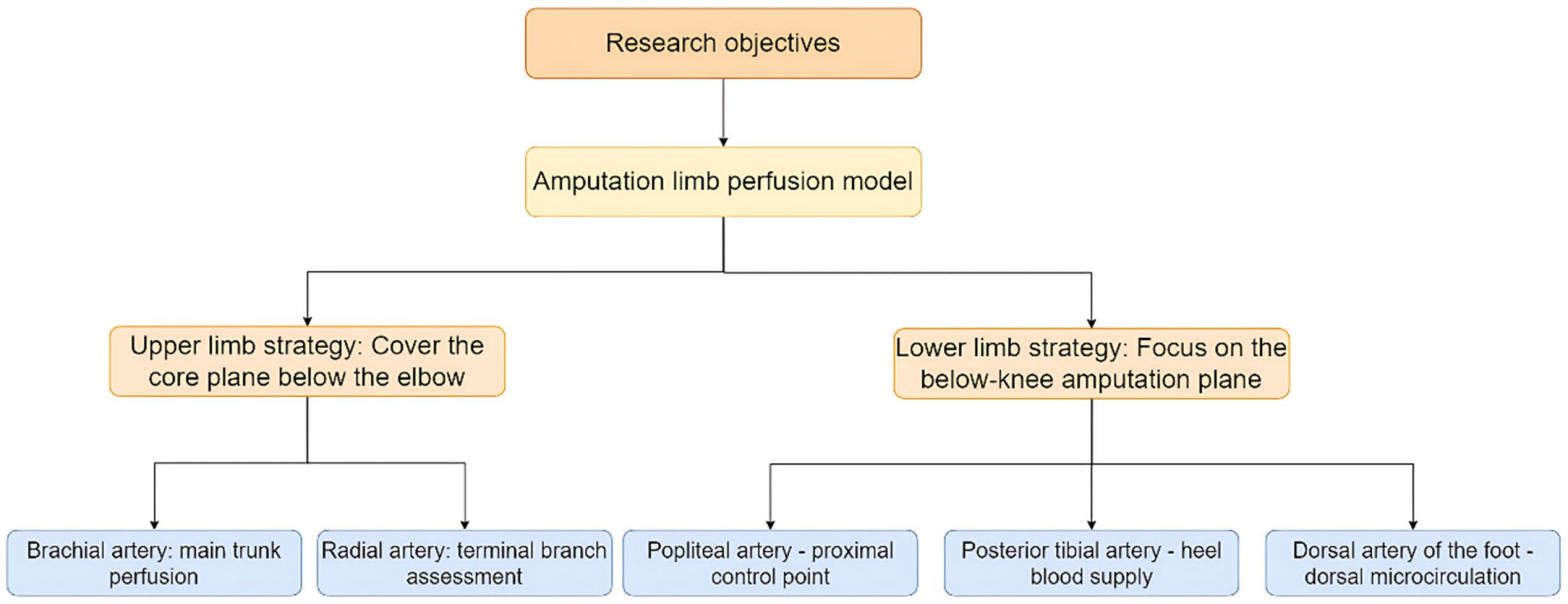
The anatomical and clinical logic chain of research design.

**Figure 2:**
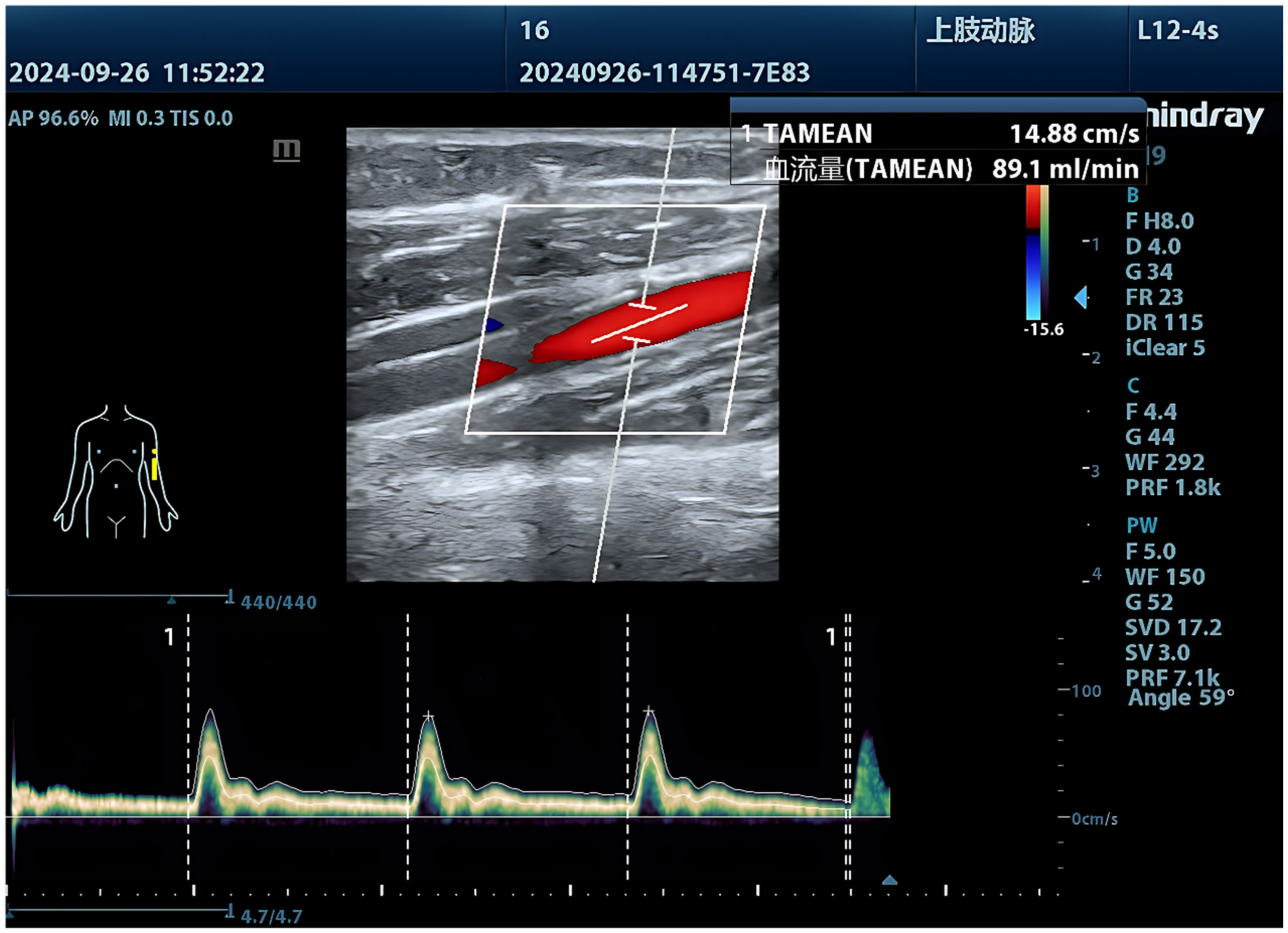
Portable color Doppler ultrasound displays arterial blood flow.

**Figure 3:**
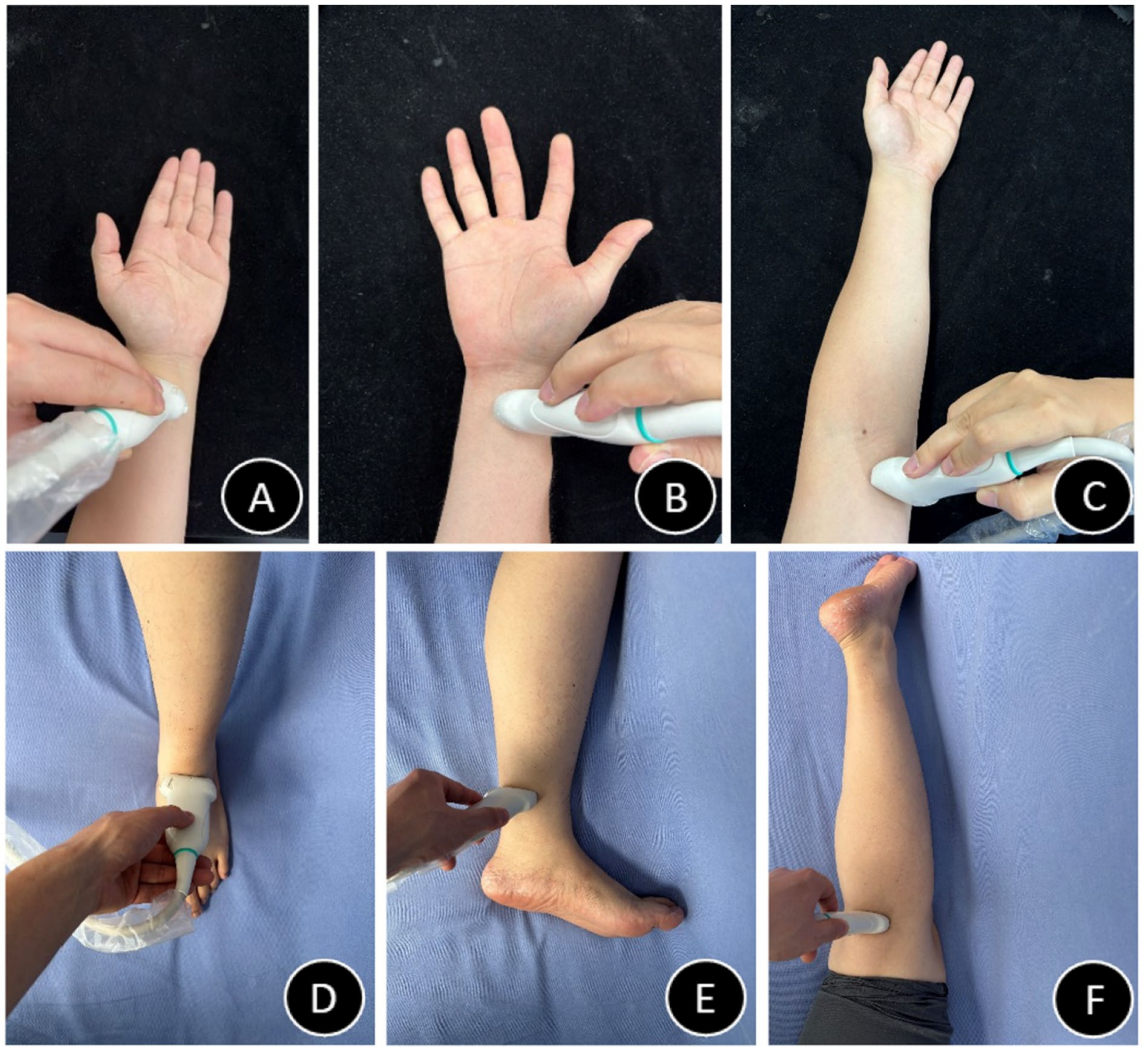
Probe positioning diagrams **(A–F)**. **(A)** Radial artery probe placement schematic. **(B)** Ulnar artery probe placement schematic. **(C)** Brachial artery probe placement schematic. **(D)** Dorsalis pedis artery probe placement schematic. **(E)** Posterior tibial artery probe placement schematic. **(F)** Popliteal artery probe placement schematic.

1. Ultrasound examination of the brachial, ulnar, and radial arteries: The exam room temperature should be maintained at 23–25°C (73–77°F). The patient rests in a seated position for 20 min before assuming the supine position. During the examination, the upper limb should be naturally extended and placed flat with the palm facing upward. The arm is positioned at heart level and moved laterally approximately 15 cm (6 inches) away from the body. Following anatomical atlas guidelines (13), the transducer was gently positioned at specified locations: ① the brachial artery measurement site 1 cm proximal to its bifurcation in the antecubital fossa, and ② the radial/ulnar artery measurement sites 2 cm proximal to the wrist crease. Using B-mode imaging, transverse views of the brachial, radial, and ulnar arteries were obtained to measure arterial diameters. The probe was then rotated 90° to longitudinal orientation with color Doppler mode to visualize luminal blood flow distribution. Pulse-wave Doppler mode was subsequently activated with the sample volume positioned at mid-lumen (adjusted to 1/2–2/3 of the luminal diameter) and Doppler angle maintained below 60° to acquire arterial spectral waveforms. Three consecutive measurements of arterial diameter, time-averaged blood flow velocity (cm/s), and volumetric flow rate (mL/min) were recorded per vessel, with averaged values calculated to ensure measurement reliability.
2. Ultrasound examination of the popliteal, dorsalis pedis, and posterior tibial arteries: The examination room temperature should be maintained at 23–25°C (73–77°F). After resting in a seated position for 20 min, the subject assumes a prone position. During the procedure, the subject lies flat on the abdomen with arms flexed and positioned alongside the head, legs fully extended. Soft cushions are placed under the chest, hips, and ankles to optimize positioning, and the head is turned to one side for comfort and airway accessibility. Following anatomical atlas guidelines (13), the transducer was positioned at defined landmarks: ① popliteal artery at the mid-popliteal fossa, ② dorsalis pedis artery at the ankle midpoint between the medial and lateral malleoli, and ③ posterior tibial artery 3–4 cm posterior-superior to the medial malleolus. B-mode imaging first captured transverse arterial cross-sections for luminal diameter measurements. The probe was then rotated 90° to longitudinal orientation, with color Doppler confirming vascular patency through luminal flow filling. Pulse-wave Doppler analysis followed, using standardized parameters: mid-lumen sample volume (1/2 to 2/3 of luminal diameter), Doppler angle <60°, and spectral waveform acquisition for hemodynamic profiling. Triplicate measurements of arterial diameter (mm), peak systolic velocity (cm/s), and volumetric flow rate (mL/min) were recorded per vessel, with cycle-averaged values derived for clinical documentation (Figures 1–3).

### Statistical analysis

Statistical analyses were performed using SPSS 27.0 software (IBM, United States). Continuous variables were initially evaluated for normality through the Kolmogorov–Smirnov test. Normally distributed data were expressed as mean ± standard deviation (SD), while non-normally distributed data were presented as median (interquartile range, IQR). Paired-sample *t*-tests were employed to compare hemodynamic differences between paired arterial branches (ulnar vs. radial arteries; dorsalis pedis vs. posterior tibial arteries). Linear regression analysis was conducted for normally distributed variables. Based on the distinct anatomical distribution patterns of traumatic limb amputations (14, 15), the brachial and popliteal arteries were selected as primary study targets. Multiple linear regression analysis models were developed for each artery, respectively. Age, sex, hypertension, diabetes, hyperlipidemia, BMI, and BSA were incorporated into the regression model using stepwise selection (entry criterion: *p* < 0.05; removal criterion: *p* ≥ 0.5). Categorical variables—sex (0 = female, 1 = male), diabetes (0 = absent, 1 = present), hypertension (0 = absent, 1 = present), and hyperlipidemia (0 = absent, 1 = present)—were dummy-coded. This yielded a predictive model for arterial blood perfusion volume. Hayes’ PROCESS macro (Model 1 for moderation analysis) was employed to investigate interaction effects. After controlling for main effects, interaction terms (e.g., mean-centered age × diabetes status) were automatically generated by including both main and interaction effects in the model. All continuous variables were mean-centered to reduce multicollinearity. Significance of interaction effects was evaluated using *t*-tests (*α* = 0.05) with 95% bias-corrected bootstrap confidence intervals (5,000 resamples).

## Result

### Baseline data

**Figure 4:**
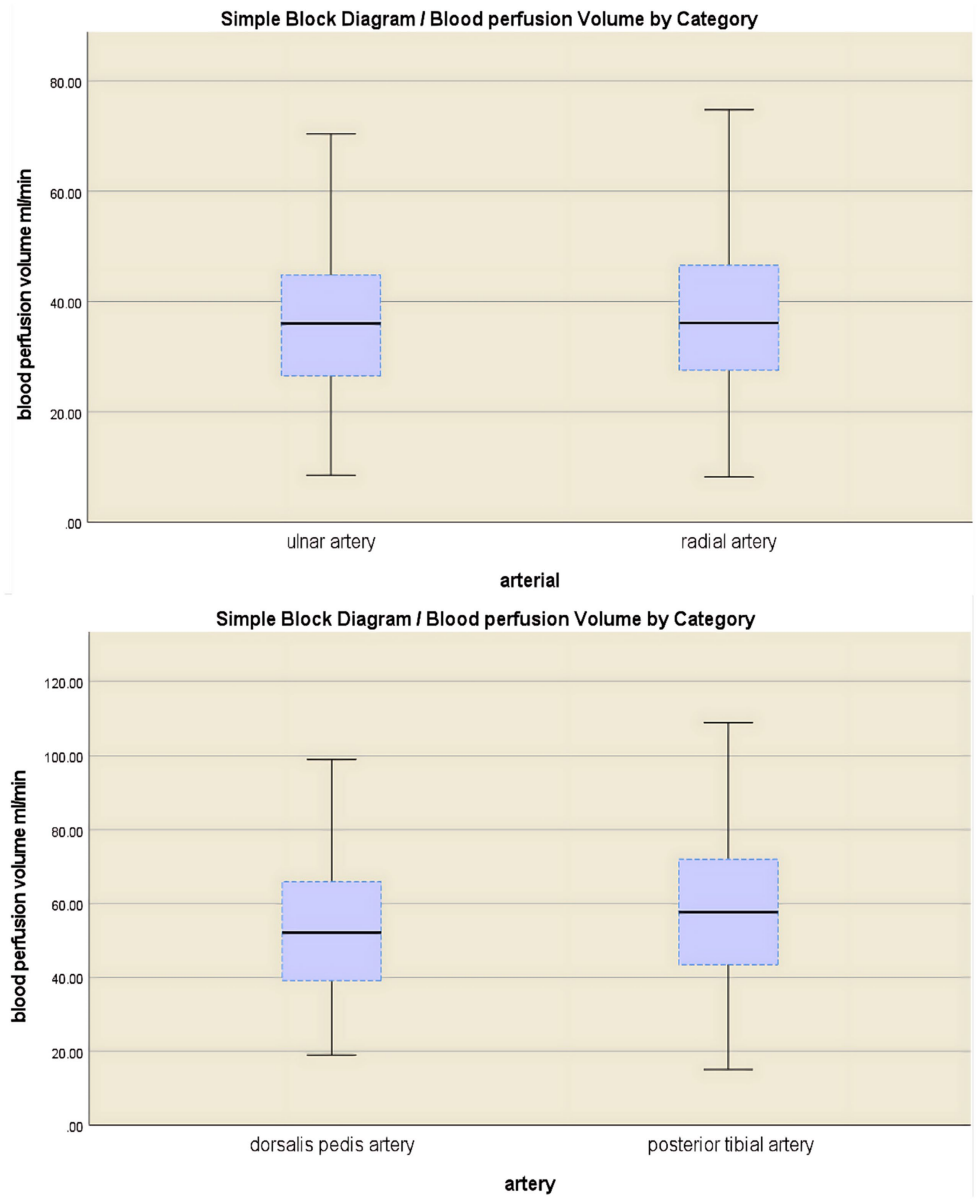
A comparative analysis of blood flow perfusion volume in branch arteries showed no significant difference between the ulnar artery and the radial artery (*p* > 0.05), while the blood flow perfusion volume in the posterior tibial artery was significantly greater than that in the dorsal artery of the foot (*p* < 0.001).

The study cohort comprised 304 participants (mean age 41.68 ± 11.28 years, range 18–65 years). A total of 1,824 arteries were analyzed. In the upper extremities, the brachial artery demonstrated a mean blood perfusion volume of 74.9 ± 22.5 mL/min, while the ulnar and radial arteries exhibited a mean blood perfusion volume of 35.7 ± 12.6 mL/min and 36.8 ± 13.5 mL/min, respectively. In the lower limb arteries, the mean blood perfusion volume was 114.1 ± 34.2 mL/min for the popliteal artery, 53.3 ± 18.1 mL/min for the dorsalis pedis artery, and 59.2 ± 21.0 mL/min for the posterior tibial artery. Among the branches of the brachial artery (upper limb), no significant difference was observed in mean blood perfusion volume between the ulnar and radial arteries (*p* > 0.05). However, the posterior tibial artery exhibited significantly higher blood perfusion volume compared to the dorsalis pedis artery (*p* < 0.001) (Figure 4 and Table 1).

**Table 1:** Luminal diameter, blood flow velocity, and blood perfusion volume by artery group: mean ± SD or median (Q1, Q3).

Arteries	Vessel diameter (cm)	Blood flow velocity (cm/s)	Blood perfusion (mL/min)	*t*	*p*
Brachial artery	0.36 ± 0.06	12.5 (8.8, 17.2)	74.9 ± 22.5		
Ulnar artery	0.27 ± 0.05	10.1 (6.9, 14.8)	35.7 ± 12.6	−1.442	0.15
Radial artery	0.28 ± 0.06	10.1 (6.7, 14.5)	36.8 ± 13.5		
Popliteal artery	0.47 ± 0.08	10.9 (8.3, 15.3)	114.1 ± 34.2		
Dorsalis pedis artery	0.27 ± 0.05	15.1 (10.8, 22.5)	53.3 ± 18.1	−7.248	<0.001
Posterior tibial artery	0.32 ± 0.06	11.9 (8.5, 16.9)	59.2 ± 21.0		

### Linear relationship in major limb arterial blood perfusion volume

#### Analysis of the brachial artery blood flow prediction model (upper limb)

Stepwise regression identified BSA and BMI as core predictors. The overall model was statistically significant (*p* < 0.001), with a standard error of estimate of 8.49 mL/min. The model accounted for 85.7% of the variance (adjusted *R*^2^ = 0.857). No other variables (age, sex, hypertension, diabetes, hyperlipidemia) met the entry criterion (all *p* > 0.05). BSA emerged as the strongest predictor (*β* = 0.637), with each 1 m^2^ increase associated with a 105.91 mL/min rise in blood flow (95% CI: 96.33–115.49). BMI demonstrated a significant negative association (*β* = −0.370), where each 1 kg/m^2^ increment corresponded to a 3.53 mL/min reduction in blood flow (95% CI: 2.978–4.077). All variance inflation factors (VIF) were <2, indicating absence of multicollinearity (Table 2). Model diagnostics revealed: Histogram of standardized residuals approximated a normal distribution (mean = −7.49 × 10^−15^, SD = 0.997). Points in the normal P–P plot largely adhered to the diagonal line, supporting normality of residuals. Residuals-versus-predicted values scatter plot showed random dispersion without discernible patterns, meeting homoscedasticity assumptions (Supplementary Data Sheet 1).

**Table 2:** Regression coefficients of the brachial artery blood flow prediction model.

Variables	*B* (95% CI)	Beta	*t*	*p*	VIF	*R* ^2^	Adjust *R*^2^	SEE
Constants	−27.937 (0.440, 56.315)	—	−1.937	0.054		0.858	0.857	8.49
BSA	105.908 (96.325, 115.491)	0.637	21.748	<0.001	1.821			
BMI	−3.527 (−4.077, −2.978)	−0.370	−12.634	<0.001	1.821			

#### Analysis of the lower limb popliteal artery blood flow prediction model

Stepwise regression identified BSA, BMI, age, and diabetes as key predictors. The overall model effect was highly significant (*p* < 0.001), with a standard error of estimate of 14.42 mL/min. The total explained variance reached 82.2% (adjusted *R*^2^ = 0.822). Other variables (sex, hypertension, hyperlipidemia) did not meet the inclusion criteria (all *p* > 0.05). Among the predictors, BSA was the strongest predictor (*β* = 0.564), with each 1 m^2^ increase associated with a 142.62 mL/min increase in blood flow (95% CI: 126.024–159.220). BMI showed a significant negative association (*β* = −0.392), with each 1 kg/m^2^ increase associated with a 5.69 mL/min decrease in blood flow (95% CI: 4.734–6.639). Each 1-year increase in age was associated with a 0.24 mL/min increase in blood flow (95% CI: 0.087–0.390; *β* = 0.079). Patients with diabetes had an average decrease of 5.60 mL/min in blood flow (95% CI: 1.783–9.427; *β* = −0.081). All variance inflation factors (VIF) were <2, indicating absence of multicollinearity (Table 3). Model diagnostics revealed: Histogram of standardized residuals approximated a normal distribution (mean = −5.71 × 10^−15^, SD = 0.993). Points in the normal P–P plot largely adhered to the diagonal line, supporting normality of residuals. Residuals-versus-predicted values scatter plot showed random dispersion without discernible patterns, meeting homoscedasticity assumptions (Supplementary Data Sheet 2).

**Table 3:** Regression coefficients of the popliteal artery blood flow prediction model.

Variables	*B* (95% CI)	Beta	*t*	*p*	VIF	*R* ^2^	Adjust *R*^2^	SEE
Constants	−8.437 (−57.027, 40.152)	—	−0.342	0.733	—	0.825	0.822	14.42
BSA	142.622 (126.024, 159.220)	0.564	16.910	<0.001	1.894			
BMI	−5.687 (−6.639, −4.734)	−0.392	−11.752	<0.001	1.897			
Age	0.238 (0.087, 0.390)	0.079	3.090	0.002	1.104			
Diabetes	−5.605 (−9.427, −1.783)	−0.081	−2.886	0.004	1.359			

Through multiple linear regression, predictive formulas for limb blood flow based on BMI index and BSA can be established. Taking the brachial artery and popliteal artery as examples: the brachial artery prediction formula is: Brachial artery blood flow = −27.937 + 105.908 × BSA − 3.527 × BMI (adjusted *R*^2^ = 0.857, SEE = 8.49 mL/min); the popliteal artery prediction formula is: Popliteal artery blood flow = −8.437 + 142.622 × BSA − 5.687 × BMI + 0.238 × age − 5.605 × diabetes (adjusted *R*^2^ = 0.822, SEE = 14.42 mL/min).

### Interaction effect analysis exploring moderating effects

Garg et al. (16) found that forearm and lower leg blood flow in both limbs was significantly reduced in the obese group. Guo et al. (17) suggested that diabetes accelerates vascular aging. Based on these physiological and metabolic pathological mechanisms, this study specifically examined interaction effects of key variables: the BSA × BMI interaction term; the age × diabetes interaction term. After establishing the main effects model, to explore synergistic mechanisms between factors, the following hypothesized interaction terms were introduced: BSA × BMI interaction: examining whether obesity status moderates the blood flow effect of BSA; age × diabetes interaction: verifying whether diabetes attenuates age-related blood flow increase.

#### Interaction effect analysis exploring moderating effects for the brachial artery blood flow prediction model

Hayes’ PROCESS Model 1 (moderating effect model) was used to analyze the impact of interaction terms of key variables (with significance) on blood flow. Key variables underwent centralized processing (BSA_c, BMI_c).

**Figure 5:**
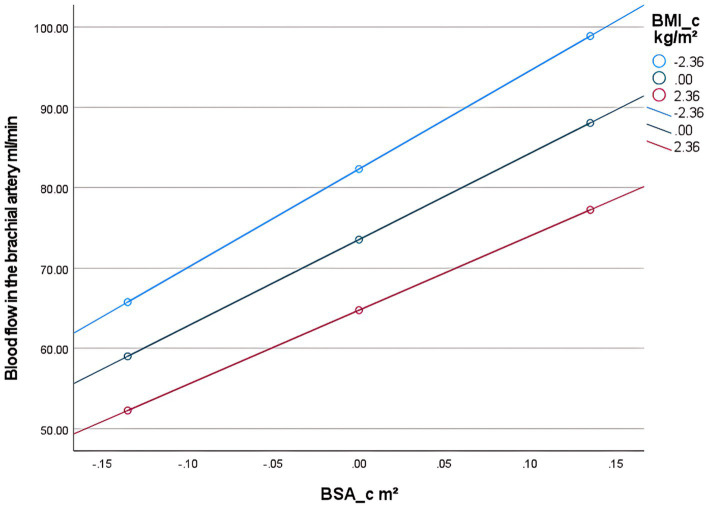
Conditional effect slope plot of BMI adjustment in the brachial artery prediction model.

The brachial artery blood flow prediction model was overall significant [*F*(3,300) = 645.82, *p* < 0.001], explaining 86.5% of the variance in brachial artery blood flow (adjusted *R*^2^ = 0.865). The interaction effect was significant: the BSA × BMI interaction term had a significant negative effect on brachial artery blood flow [*β* = −6.39, *p* < 0.001, 95% CI (−9.45, −3.33)]. The interaction term explained an incremental variance of 0.8% [Δ*R*^2^ = 0.008, Δ*F*(1,300) = 16.87, *p* < 0.001], indicating that BMI significantly moderated the relationship between BSA and blood flow. Simple slope analysis: Under different BMI levels, the strength of the positive effect of BSA on blood flow showed significant differences: Low BMI group (1 SD below mean, BMI_c = −2.36): Every 1 m^2^ increase in BSA increased blood flow by 122.51 mL/min [SE = 6.23, *p* < 0.001, 95% CI (110.24, 134.78)]. Average BMI group (BMI_c = 0): Every 1 m^2^ increase in BSA increased blood flow by 107.45 mL/min [SE = 4.76, *p* < 0.001, 95% CI (98.08, 116.82)]. High BMI group (1 SD above mean, BMI_c = +2.36): Every 1 m^2^ increase in BSA increased blood flow by 92.39 mL/min [SE = 5.78, *p* < 0.001, 95% CI (81.02, 103.75)] (Table 4). The blood flow gain effect in high-BMI individuals decreased by 14.0% compared to normal-BMI individuals [(107.45–92.39)/107.45], and by 24.6% compared to low-BMI individuals [(122.51 to 92.39)/122.51] (Table 5). Moderation direction: Increased BMI significantly weakened the positive promoting effect of BSA on brachial artery blood flow (Figure 5). BMI negatively moderated the relationship between BSA and brachial artery blood flow: as BMI increased, the positive promoting effect of BSA on blood flow significantly attenuated. The standardized residual histogram showed an approximately normal distribution; in the P–P plot, observed points were distributed essentially along the diagonal, indicating residuals conformed to the normality assumption; the residual-predicted value scatter plot showed randomly distributed residuals without significant heteroscedasticity patterns, satisfying the homoscedasticity assumption (Supplementary Data Sheet 3).

**Table 4:** Overall regression model results after introducing the interaction term (BSA × BMI) (prediction of brachial artery blood flow).

Variables	*β* (SE)	*t*	*p*	95% CI	Δ*R*^2^	Δ*F*
Constant	73.54 (0.58)	126.99	<0.001	(72.40, 74.68)	0.008	16.87
BSA	107.45 (4.76)	22.57	<0.001	(98.08, 116.82)		
BMI	−3.73 (0.28)	−13.48	<0.001	(−4.27, −3.18)		
Interaction item (BSA × BMI)	−6.39 (1.55)	−4.11	<0.001	(−9.45, −3.33)		
Model overview: *R*^2^ = 0.865, *F*(3,300) = 645.82, *p* < 0.001						

**Table 5:** Conditional effects under BMI adjustment in the brachial artery prediction model (effect of BSA on blood flow).

BMI level	Actual BMI	Effect value (SE)	*t*	*p*	95% CI	Effect intensity variation
Low BMI	Mean − 1SD	122.51 (6.23)	19.65	<0.001	(110.24, 134.78)	+14.0%↑
Average BMI	Sample mean	107.45 (4.76)	22.57	<0.001	(98.08, 116.82)	Reference benchmark
High BMI	Mean + 1SD	92.39 (5.78)	15.99	<0.001	(81.02, 103.75)	−14.0%↓

#### Interaction effect analysis exploring moderating effects for the popliteal artery blood flow prediction model

Hayes’ PROCESS Model 1 (moderating effect model) was used to analyze the impact of interaction terms of key variables (with significance) on blood flow. Key variables underwent centralized processing (BSA_c, BMI_c, Age_c).

First, examining the moderating effect of BMI on the relationship between BSA and popliteal artery blood flow, controlling for the influence of age and diabetes history. The regression model was overall significant [*F*(5,298) = 305.33, *p* < 0.001], explaining 83.67% of the variance in popliteal artery blood flow (*R*^2^ = 0.8367). Key results are as follows: The interaction effect was significant: the BSA × BMI interaction term had a significant negative effect on popliteal artery blood flow [*β* = −12.32, SE = 2.63, *p* < 0.001, 95% CI (−17.50, −7.15)]. The interaction term explained an additional 1.2% of the variance [Δ*R*^2^ = 0.012, Δ*F*(1,298) = 21.99, *p* < 0.001], indicating that BMI significantly moderated the relationship between BSA and popliteal artery blood flow (Table 6). Simple slope analysis: Low BMI group (1 SD below mean, BMI = mean −2.36): Every 1-unit (m^2^) increase in BSA significantly increased popliteal artery blood flow by 175.28 mL/min [SE = 10.72, *p* < 0.001, 95% CI (154.18, 196.38)]. Average BMI group (BMI = sample mean): Every 1-unit increase in BSA increased blood flow by 146.22 mL/min [SE = 8.19, *p* < 0.001, 95% CI (130.10, 162.33)]. High BMI group (1 SD above mean, BMI = mean +2.36): Every 1-unit increase in BSA increased blood flow by only 117.16 mL/min [SE = 9.80, p < 0.001, 95% CI (97.88, 136.43)]. The blood flow gain effect in high-BMI individuals decreased by 19.9% compared to average-BMI individuals [(146.22 to 117.16)/146.22 × 100%], and by 33.2% compared to low-BMI individuals [(175.28 to 117.16)/175.28 × 100%] (Table 7). Moderation direction: Increased BMI significantly weakened the positive effect of BSA on popliteal artery blood flow (Figure 5). Covariate effects: Age showed a weak positive correlation with popliteal artery blood flow (*β* = 0.24, *p* = 0.002); diabetes significantly reduced blood flow (*β* = −4.89, *p* = 0.01). High BMI reduced the blood flow-promoting effect of BSA by nearly 20%, suggesting obesity may impair the physiological compensatory capacity of lower limb vasculature. The standardized residual histogram showed an approximately normal distribution; in the P–P plot, observed points were distributed essentially along the diagonal, indicating residuals conformed to the normality assumption; the residual-predicted value scatter plot showed randomly distributed residuals without significant heteroscedasticity patterns, satisfying the homoscedasticity assumption (Supplementary Data Sheet 4).

**Table 6:** Overall regression model results after introducing the interaction term (BSA × BMI) (prediction of popliteal artery blood flow).

Variables	*β* (SE)	*t*	*p*	95% CI	Δ*R*^2^	Δ*F*
Constants	103.82 (3.14)	33.01	<0.001	(97.63, 110.00)	0.012	21.99
BSA	146.22 (8.19)	17.86	<0.001	(130.10, 162.33)		
BMI	−6.11 (0.48)	−12.82	<0.001	(−7.04, −5.17)		
BSA × BMI	−12.32 (2.63)	−4.69	<0.001	(−17.50, −7.15)		
Age	0.24 (0.07)	3.17	0.002	(0.09, 0.38)		
Diabetes	−4.89 (1.88)	−2.60	0.010	(−8.60, −1.19)		
Overall model: *R*^2^ = 0.8367, *F*(5,298) = 305.33, *p* < 0.001						

**Table 7:** Conditional effects under BMI adjustment in the popliteal artery prediction model (effect of BSA on blood flow).

BMI level	Actual BMI	Effect value (SE)	*t*	*p*	95% CI	Effect intensity variation
Low BMI	Mean − 1SD	175.28 (10.72)	16.35	<0.001	(154.18, 196.38)	+19.9%↑
Average BMI	Sample mean	146.22 (8.19)	17.86	<0.001	(130.10, 162.33)	Reference benchmark
High BMI	Mean + 1SD	117.16 (9.80)	11.96	<0.001	(97.88, 136.43)	−19.9%↓

**Figure 6:**
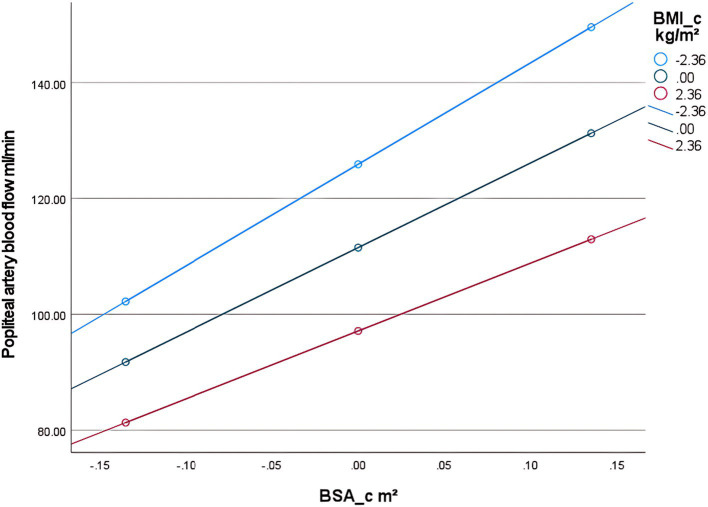
Conditional effect slope plot of BMI adjustment in the popliteal artery prediction model.

**Figure 7:**
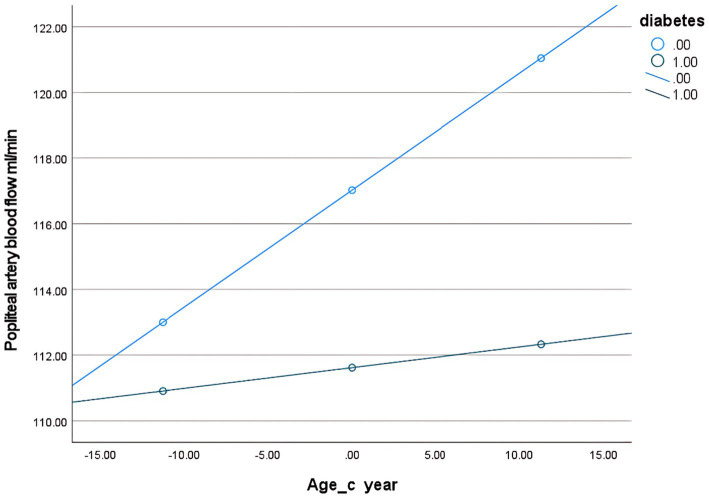
Conditional effect slope plot of diabetes regulation in the popliteal artery prediction model.

Second, to examine the moderating effect of diabetes on the relationship between age and popliteal artery blood flow, controlling for covariates. The overall regression model was significant [*F*(5, 298) = 284.24, *p* < 0.001], explaining 82.67% of the variance in popliteal artery blood flow (*R*^2^ = 0.8267). The key results were as follows: The age-by-diabetes interaction term on popliteal artery blood flow was not significant [*β* = −0.29, SE = 0.16, *p* = 0.063, 95% CI (−0.60, 0.02)]. There is insufficient evidence to conclude that the relationship between age and blood flow differs significantly between the “with diabetes” and “without diabetes” groups. In this model, age is an independent predictor of blood flow, with popliteal artery blood flow increasing by an average of 0.36 mL/min for each 1-year increase in age. BSA (*B* = 142.17, *p* < 0.001) and BMI (*B* = −5.65, *p* < 0.001) were both strong predictors of blood flow. Diabetes independently reduced blood flow by 5.41 mL/min (*p* = 0.006) (Table 8). Simple slope analysis revealed that popliteal artery blood flow consistently increased with age in the non-diabetic group, whereas it remained relatively stable in the diabetic group. However, this difference did not reach statistical significance (Figure 6). The histogram of the model’s standardized residuals demonstrated an approximately normal distribution. In the P–P plot, the observed points largely followed the diagonal line, suggesting the residuals met the assumption of normality. The residual-versus-predicted values scatter plot showed randomly distributed residuals with no clear heteroscedasticity pattern, satisfying the assumption of homoscedasticity (Supplementary Data Sheet 5) (see Figure 7).

**Table 8:** Overall regression model results (prediction of popliteal artery blood flow) after introducing interaction terms (age × diabetes).

Variables	*β* (SE)	*t*	*p*	95% CI	Δ*R*^2^	Δ*F*
Constants	1.80 (24.43)	0.07	0.941	(−46.28, 49.88)	0.002	3.49
Age	0.36 (0.10)	3.58	0.0004	(0.16, 0.55)		
Diabetes	−5.41 (1.94)	−2.79	0.006	(−9.22, −1.60)		
Age × Diabetes	−0.29 (0.16)	−1.87	0.063	(−0.60, 0.02)		
BMI	−5.65 (0.48)	−11.71	<0.001	(−6.60, −4.70)		
BSA	142.17 (8.40)	16.92	<0.001	(125.63, 158.70)		
Overall model: *R*^2^ = 0.8267, *F*(5, 298) = 284.24, *p* < 0.001						

## Discussion

In organ transplantation, the precise regulation of perfusion parameters is a critical factor in ensuring the viability of transplanted organs. In cardiac transplantation, normothermic blood perfusion volumes are typically maintained between 650–850 mL/min to preserve coronary microcirculation patency and prevent endothelial injury. In *ex vivo* lung perfusion (EVLP) (18–20), a blood perfusion volume of 150 mL/min is typically utilized to mitigate pulmonary vascular bed pressure and maintain alveolar oxygenation efficiency (21, 22). In renal transplantation, the blood perfusion volume is generally maintained within a range of 300–500 mL/min to balance metabolic substrate delivery and mitigate the risk of cellular edema (23, 24). In contrast, determining blood perfusion volume for limb perfusion (e.g., in *ex vivo* limb preservation or composite tissue transplantation) presents more complex challenges: Limbs are composed of multiple tissue types—including muscle (high metabolic demand), nerves (ischemia-sensitive), bone (low metabolic demand), and skin—exhibiting marked metabolic heterogeneity. At rest, skeletal muscle blood flow is relatively low; however, post-reperfusion, it may surge abruptly, potentially leading to muscle hyperperfusion, which triggers edema and subsequent compartment syndrome, resulting in irreversible damage (11). This complexity underscores the necessity for personalized blood perfusion volume settings in limb preservation. Optimal perfusion parameters are not only critical for graft survival but also directly influence postoperative functional recovery and the regulation of rejection responses, representing a pivotal breakthrough in transitioning limb replantation (or transplantation) from technical feasibility to routine clinical practice. Within the scope of the reviewed literature, this study is the first to investigate blood perfusion volume in the major arteries of the extremities in healthy populations, limb blood perfusion volume at different amputation levels, and the correlation between limb blood perfusion volume and commonly used clinical indices such as BMI and BSA.

### Normal physiology: extremity artery perfusion anatomy and measurement variability

This study measured blood flow in the major arteries of the limbs at different levels, revealing characteristic patterns of post-branching hemodynamic changes within the arterial system. In the upper extremity vascular system, blood flow from the brachial artery (cubital fossa level) to the ulnar and radial arteries (wrist joint level) consistently showed a decrease of approximately 48% (74.9 mL/min vs. 35.7–36.8 mL/min). In contrast, the decrease in blood flow from the popliteal artery (popliteal fossa level) to the dorsalis pedis and posterior tibial arteries (ankle joint level) in the lower extremity exhibited marked differences (114.1 mL/min vs. 53.3–59.2 mL/min). Based on Hagen–Poiseuille’s law, it is inferred that after arterial branching, the cross-sectional area of the branch arteries decreases, while peripheral resistance increases (25). This aligns with the distal artery blood flow decrease phenomenon observed in the present study. It should be noted that no statistically significant difference was found in perfusion volume between the ulnar and radial arteries at the same transection level (*p* > 0.05); this finding is consistent with results from anatomical studies of these arteries (26). In contrast, the mean perfusion volume of the posterior tibial artery was greater than that of the dorsalis pedis artery (*p* < 0.001). This finding also aligns with anatomical studies demonstrating a larger diameter in the posterior tibial artery compared to the dorsalis pedis artery (27, 28). This suggests the potential existence of more complex blood flow distribution mechanisms in the distal lower extremity, which may be related to the physiological demands of the plantar vascular network. Liang et al. (29, 30) measured mean blood perfusion volumes in the popliteal and dorsalis pedis arteries using Doppler ultrasound, reporting values of 92.1 mL/min and 33.0 mL/min, respectively—results consistent with those of the present study. Klein et al. (31), employing magnetic resonance imaging (MRI) to assess peripheral arterial blood perfusion in healthy individuals, documented a mean popliteal artery blood perfusion volume of 61.9 mL/min differs significantly from this study. These discrepancies highlight the potential impact of measurement device variability on outcomes. Future efforts should prioritize developing AI-driven analytical tools and software to enhance the precision, standardization, and accessibility of blood perfusion volume estimation in amputated limbs during *ex vivo* perfusion. Additionally, integrating ultrasound with advanced imaging modalities (e.g., MRI, CT) could provide more accurate assessments of post-replantation perfusion dynamics, thereby improving clinical decision-making in limb salvage protocols.

### Impact analysis of BMI and BSA on limb blood perfusion volume

This study employed multiple linear regression modeling to examine the effects of variables including BSA, BMI, sex, age, hypertension, diabetes, and hyperlipidemia on blood flow. Multiple linear regression analysis revealed linear relationships between both BMI and BSA with limb blood flow. Specifically, blood perfusion volume decreased with increasing BMI, while it increased with higher BSA. Elevated BMI is associated with visceral fat accumulation (32), a phenomenon that not only contributes to local tissue hypoperfusion but also exacerbates peripheral vascular resistance through systemic inflammatory responses and endothelial dysfunction. Specifically, elevated BMI levels demonstrate a significant correlation with abnormal increases in pro-inflammatory cytokines such as IL-6 and TNF-α. These cytokines play a key role in inflammatory states, contributing to vascular endothelial layer damage that ultimately compromises peripheral vascular function and elevates hemodynamic resistance (33–35). This negative regulatory mechanism accounts for the significant inverse correlation between BMI and blood perfusion volume in extremity arteries. On the other hand, a larger BSA typically reflects greater metabolic demands and elevated cardiac output (36, 37), which drives increased blood perfusion. Petrini et al. (38) demonstrated that pulmonary blood perfusion volume significantly increases with higher BSA, suggesting a close linkage between BSA and vascular functional status. The development of an arterial blood flow prediction model provides a quantitative tool for limb blood flow assessment. For example, for a 50-year-old normal patient with a BMI of 22.92 kg/m^2^ and a BSA of 1.87 m^2^, the target brachial artery blood flow and popliteal artery blood flow are 89.27 mL/min and 134.22 mL/min, respectively.

Particularly in replantation (transplantation) surgery for severed limbs, this model can assist surgeons in predicting limb perfusion volume during extracorporeal preservation at different transection levels, complementing the angiosome theory proposed by Taylor and Palmer (39).

### Assessing moderating effects in linear regression modeling

To further investigate the interactive effects of significant variables (BSA × BMI, age × diabetes) in the multiple linear regression model on blood flow and elucidate the complex mechanisms underlying blood flow regulation, this study conducted additional analyses. Regarding BMI and BSA, it was found that the interaction term between BSA and BMI (BSA × BMI) exerted a significant effect on blood flow.

Further conditional process analysis using PROCESS revealed that the effect of BSA on blood flow was significantly contingent on BMI levels. Specifically, elevated BMI attenuated the promoting effect of BSA on blood flow by nearly 15–20%. This finding highlights BMI’s pivotal moderating role in the coupling relationship between body surface area (BSA) and blood flow: Elevated BMI significantly attenuates the positive promoting effect of BSA on arterial blood flow. The underlying physiological mechanism may involve: Tissue metabolic activity differentials: Skeletal muscle exhibits significantly higher metabolic rates than adipose tissue due to its abundant mitochondrial content (40). Consequently, when increased BSA predominantly results from adipose accumulation, the resulting increment in actual oxygen consumption will be smaller than that associated with lean mass gain, thereby attenuating the physiological demand for elevated blood flow. Obesity-related metabolic dysfunction: Obesity is commonly associated with insulin resistance, which impairs tissue glucose uptake capacity and reduces overall tissue metabolic activity (41). This may attenuate metabolic signaling for blood flow demand from tissues, consequently leading to reduced arterial blood flow. When increased BSA primarily reflects adipose tissue expansion, the per-unit-BSA effective blood flow tends to decrease. Unlike the context-dependent modulation of BSA-blood flow relationships by BMI, the influences of age and diabetes demonstrate relative static independence. The study revealed that the effects of age and diabetic status on blood flow manifested primarily as significant independent main effects, with their interaction (age × diabetes) being statistically non-significant (*p* > 0.05). This indicates that the combined impact of aging and diabetes on blood flow aligns more closely with a simple additive model rather than a synergistic model. The additional hemodynamic impairment attributable to diabetes remained relatively constant across different age strata. Notably, within the diabetic population, the age-associated increase in blood flow demonstrated neither abnormal diminishment nor enhancement. This finding exhibits incomplete concordance with the hypothesis proposed by Assar et al. (42) that chronic low-grade inflammation during aging facilitates diabetes-driven vascular injury. This suggests that diabetes-related microangiopathy may follow a distinct pathological trajectory, warranting further investigation in future studies. Based on the findings of this study, predicting individual blood flow does not require additional consideration of the “age-diabetes interaction combination” (such as the amplified risk associated with the specific “older adults with diabetes” subgroup). Age and diabetic status can be incorporated as independent risk factors in predictive models. However, in obese populations, blood flow prediction models may substantially overestimate actual perfusion. For accurate assessment, models should not only include BMI and BSA as independent risk factors but must jointly incorporate their interaction term (BMI × BSA) to achieve personalized blood flow prediction.

## Limitations and future directions

This study has several limitations. First, its cross-sectional design precludes causal inferences. The single-center sampling may limit the generalizability of the findings. The relatively small sample size could potentially lead to inadequate adjustment for age stratification among diabetic participants and might obscure the impact of hyperlipidemia on blood flow. Meanwhile, the use of stepwise regression to determine the final predictive model, while useful for exploratory variable screening in the absence of a strong prior theoretical basis, carries statistical limitations: Model selection may overfit the data, reducing predictive performance for new samples (generalizability); repeated testing distorts *p*-values for variable inclusion, increasing the risk of selecting spuriously correlated variables. Therefore, despite high internal model fit in this study (adjusted *R*^2^ > 0.8), its clinical generalization should be interpreted with caution. Second, the core variable blood flow was measured using Doppler ultrasound. Although widely applied with standardized procedures, its accuracy is constrained by operator-dependent probe placement, angle correction (errors increase sharply when *θ* > 60°), subjectivity in measuring vascular diameter and tracing spectral waveforms; flow calculations assume laminar flow and axisymmetric velocity profiles, potentially introducing bias during turbulent flow or in non-circular vessels, and different instruments/settings may yield divergent results. Notwithstanding our implementation of error-control measures—including examinations by senior sonographers, utilization of standardized equipment, and averaging of repeated measurements—these inherent limitations may still introduce measurement errors that compromise the precision of the regression model estimates. Furthermore, although the model incorporated key predictors, unmeasured confounding factors (e.g., diet, genetic factors, smoking, inflammatory markers) may persist, potentially limiting its universal applicability. Notably, the intercept term indicates non-zero predicted blood flow when BSA or BMI equals zero, contradicting physiological reality. Since BSA or BMI cannot physiologically reach zero in actual scenarios, this intercept should be interpreted as a mathematical fitting result within the data range rather than a reflection of true biological relationships. Future research should focus on enhancing methodological reliability: in blood flow measurement, more advanced techniques (e.g., three-dimensional ultrasound/vector flow imaging) may be employed to reduce angle-dependent errors, and more robust analytical methods should be explored to address physiological variations; in predictive model construction, a broader range of data should be incorporated to enhance generalizability. Nonlinear approaches (e.g., piecewise regression) should be explored to capture complex relationships, while additional critical predictors should be integrated to improve model precision and utility. Alternatively, physiology/clinical knowledge-based strategies—such as hierarchical regression for testing predefined theoretical frameworks—may be employed. Modern regularization techniques like Lasso or Ridge regression could yield more robust outcomes when addressing multicollinearity and feature selection. Concurrently, larger-scale validation studies should be conducted. We plan to undertake follow-up multicenter collaborative research, ensuring adequate statistical power (target power ≥80%) across all subgroups to derive reliable conclusions.

## Conclusion

*Ex vivo* perfusion of amputated limbs is critical for improving replantation success rates, reducing ischemia-reperfusion injury, enhancing microcirculation and metabolic function, and minimizing postoperative complications, serving as a cornerstone for ensuring tissue survival and functional recovery. However, due to the lack of personalized limb hemodynamics reference standards, current clinical perfusion protocols remain challenging to tailor precisely, significantly impacting treatment outcomes. Current literature reviews reveal that while the importance of *ex vivo* perfusion for amputated limbs has received extensive attention, studies on personalized optimization of perfusion parameters during such procedures remain scarce. This study establishes arterial blood perfusion volume across various limb regions in the general population and employs linear regression analysis to elucidate the impact of BMI and BSA on extremity arterial perfusion, revealing their linear relationships. The multivariable linear regression model, constructed using key indicators such as BMI and BSA, demonstrates significant practical value in evaluating perfusion parameters for *ex vivo* preservation of amputated limbs. By integrating the linear relationships between BMI, BSA, and limb blood flow while accounting for gender, age, and metabolic factors (hypertension, diabetes, hyperlipidemia), this framework aids clinicians in estimating individualized perfusion rates. Improved perfusion accuracy during limb preservation. This approach ensures metabolic demands of amputated tissues are met during perfusion while mitigating adverse effects of over-perfusion or under-perfusion on replantation outcomes.

## Data Availability

The raw data supporting the conclusions of this article will be made available by the authors, without undue reservation.
