# Supplementary material for: Clinical study on basal blood perfusion in the major arteries of the limbs
Source: Front Med (Lausanne). 2025 Jul 30;12:1597404. doi: 10.3389/fmed.2025.1597404 (PMC12343593; doi:10.3389/fmed.2025.1597404)
Supplement: Supplementary file 1 [file Data_Sheet_1.docx]

Attachment 1


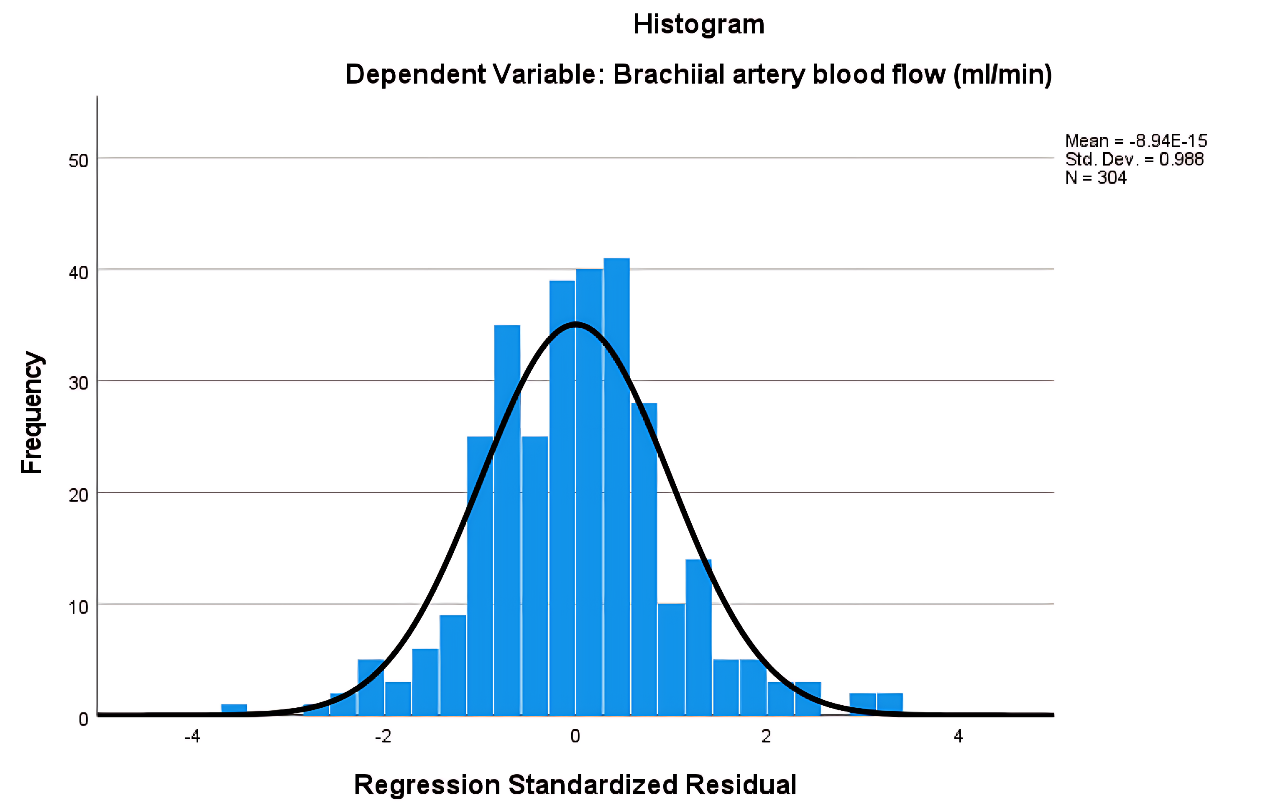


Figure 1 The standardized residual histogram shows an approximately normal distribution
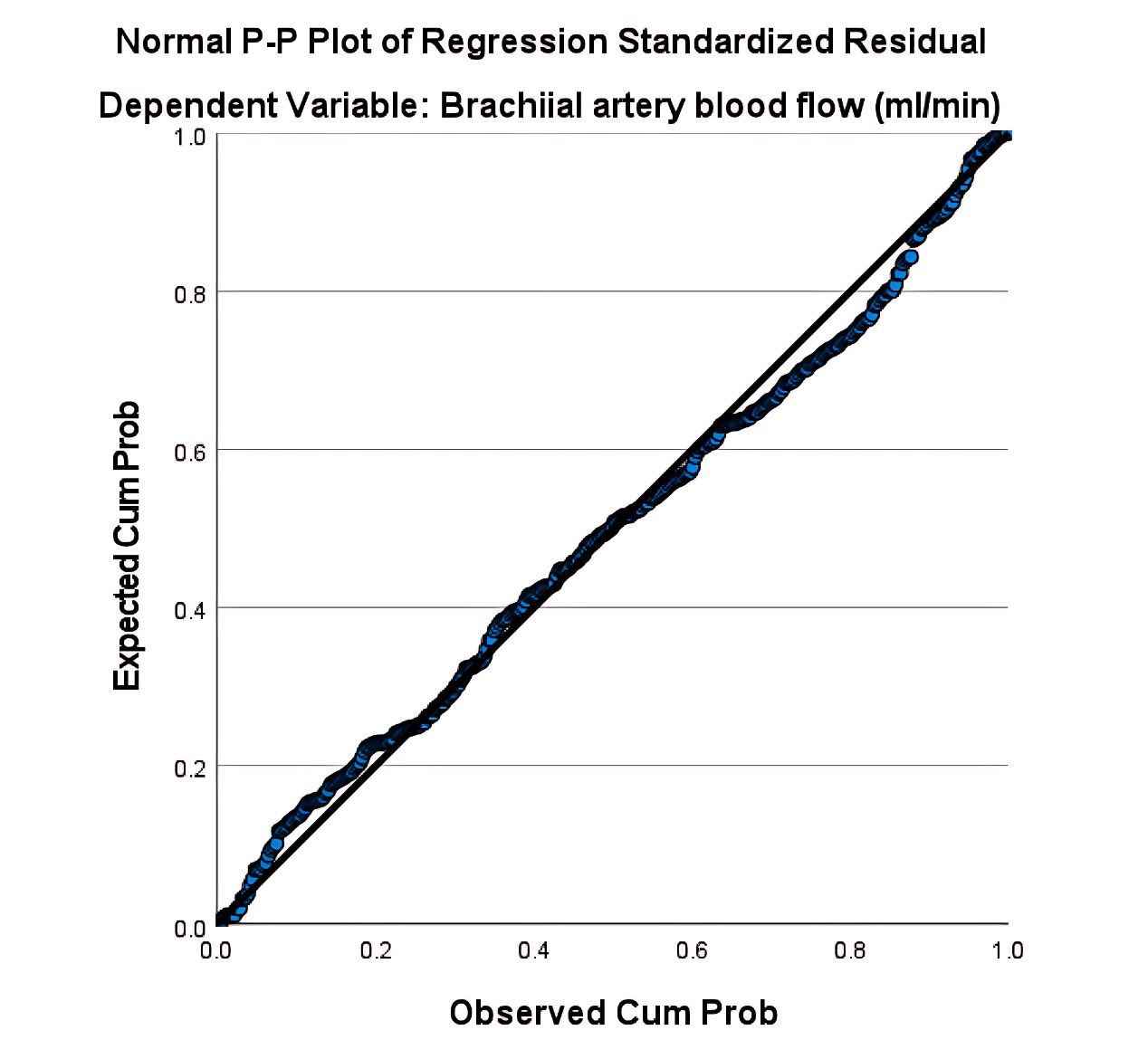


Figure 2 The observation points in the P-P plot are basically distributed along the diagonal, indicating that the residuals satisfy the normality assumption.


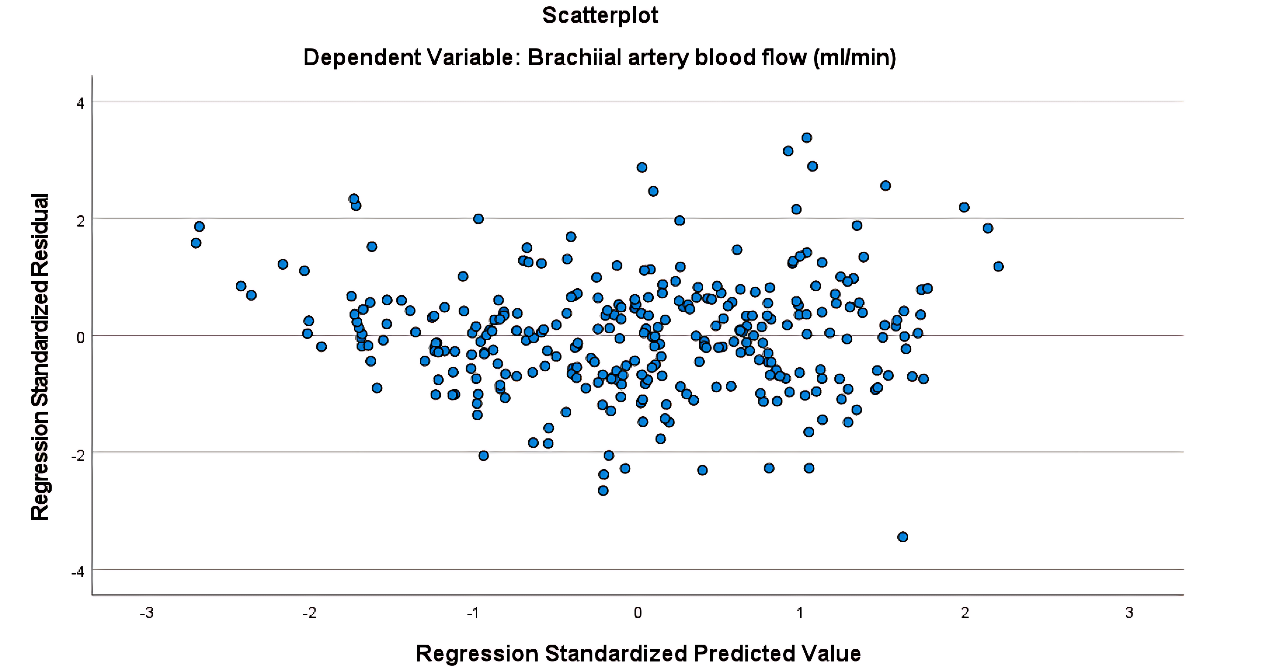


Figure 3 The residual-predicted value scatter plot shows that the residuals are randomly distributed with no obvious pattern, satisfying the assumption of homogeneity of variance.
