# Supplementary material for: Clinical study on basal blood perfusion in the major arteries of the limbs
Source: Front Med (Lausanne). 2025 Jul 30;12:1597404. doi: 10.3389/fmed.2025.1597404 (PMC12343593; doi:10.3389/fmed.2025.1597404)
Supplement: Supplementary file 4 [file Data_Sheet_4.docx]

Attachment 4


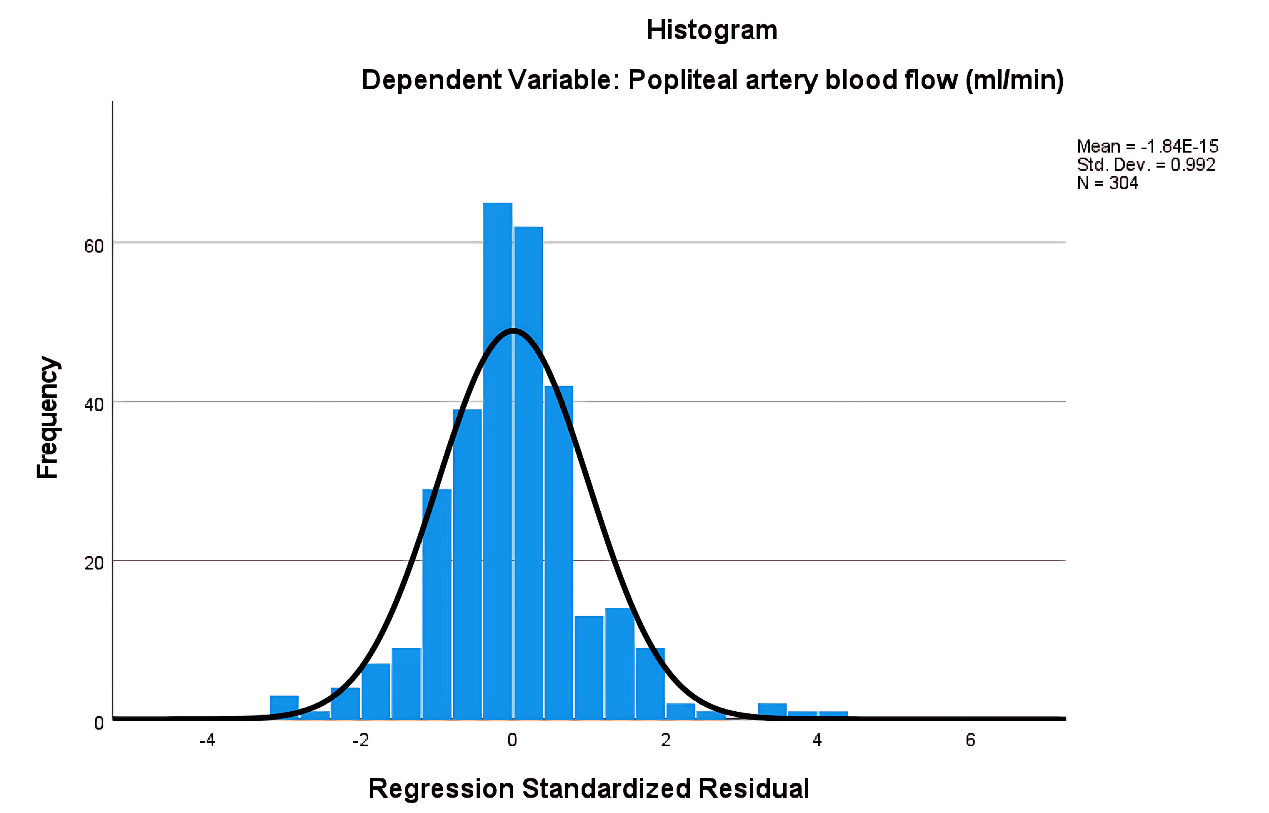


Figure 1 Standardized residual histogram shows approximate normal distribution


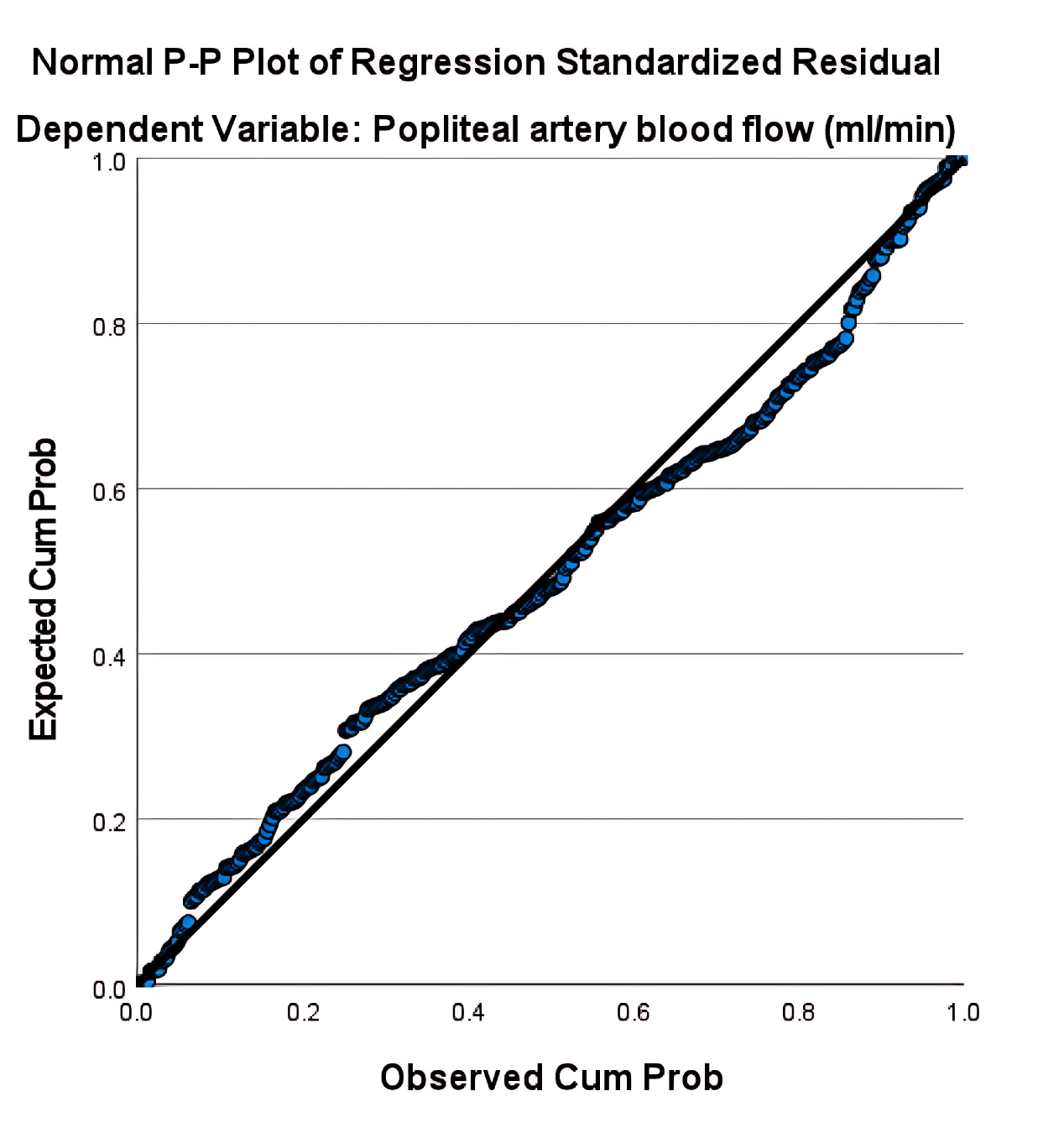


Figure 2 The observation points in the P-P plot are basically distributed along the diagonal, indicating that the residuals satisfy the normality assumption.


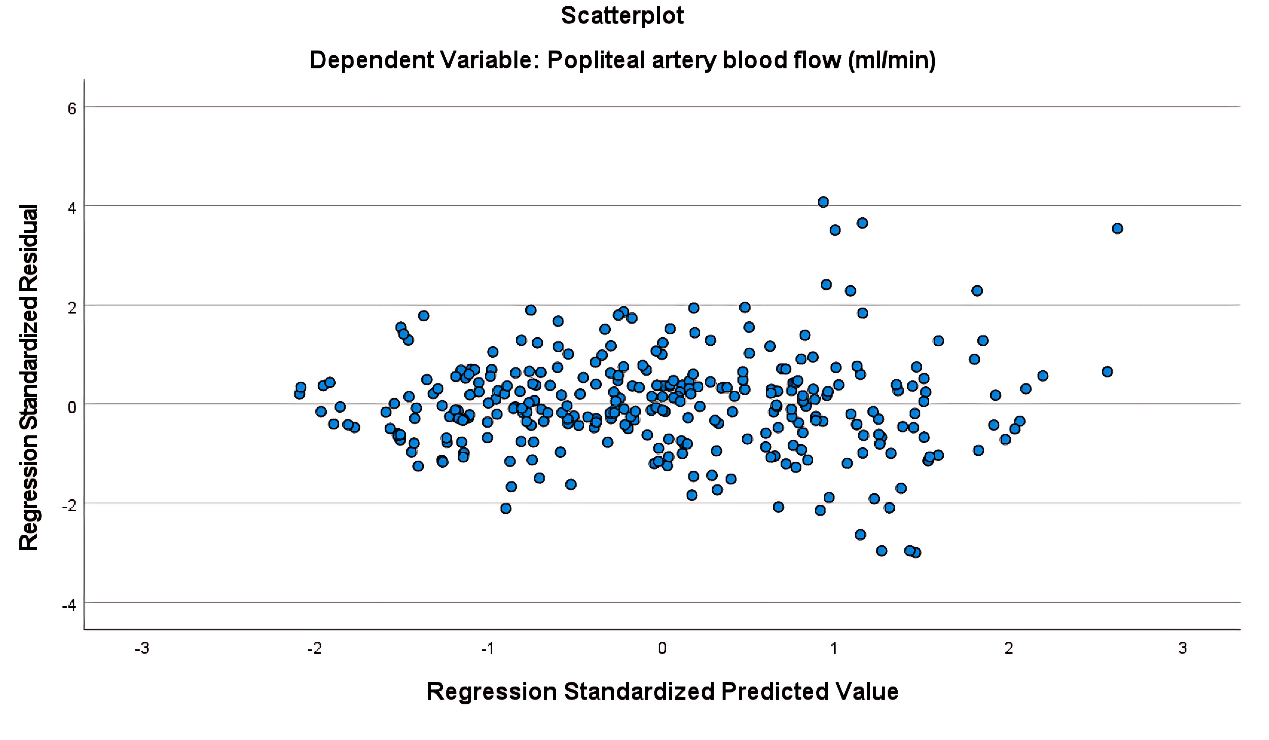


Figure 3 The residual-predicted value scatter plot shows that the residuals are randomly distributed, with no obvious heteroscedasticity pattern, satisfying the assumption of homoscedasticity.
